# Other People's Children

**Published:** 1894-01-06

**Authors:** 


### Studies in Applied Sociology.

### I.—OTHER PEOPLE'S CHILDREN.

Whatever one may think of compulsory education in general, one is forced to admit that there are some children who cannot be left to the unaided wisdom and resources of their family. Defective children, for example, require special training which it is often beyond their parents' means to procure them. Hence arise our asylums for the blind and deaf and dumb; institutions usually the offspring of charity to begin with, but brought under State supervision, and sometimes receiving State aid, as the public conscience awakes to the fact that the nation is bound to do the best it can for all its members. So, too, have sprung up our reformatory and industrial schools, which attempt the onerous task of transforming the offspring of tramps and criminals, often criminal themselves, into useful and industrious citizens.

That in all such schools the work is difficult, thankless, and often apparently futile, may be taken for granted; but it is a matter of the first importance that it should be done at all. For if it prevents even a small percentage of the defective from becoming paupers—if it saves children with criminal instincts and habits from filling our prisons, it has an inestimable economic and moral value. But, on the economic side, one should not at first demand too much. The intelligence and concentration of the blind have made them learn readily various trades in which touch and hearing are of more importance than eyesight. Blind asylums are self-supporting to a larger extent than almost any other charitable institution. But this has caused managers and teachers to expect much of their pupils; and we have known a case in which a lad who —more perhaps through neglect in childhood than through natural deficiency—seemed duller than his class-mates, being dismissed from an asylum because "he could learn nothing." This is a pernicious result of looking for immediate results in an institution which should primarily seek to confer benefits rather than to earn profits.

The methods of teaching the blind and the industries they can best learn are now almost settled; the education of the deaf and dumb is still in something of a transition state. The old method of conversing by means of the fingers is a little discounted in favour of lip-reading, and the development of the organs of speech. Anything that puts deaf mutes on an equal footing with their fellows is much to be desired; but it may be questioned if the new methods always effect this. The value of lip-reading, especially for those who have lost their hearing after they have learned to speak, is very great. Given only light, the lip reader is as capable a man as his neighbour. He requires no special training on the part of the person he is conversing with, so his circle is not limited like those who know only the finger language; his well-trained eyes serve for ears as well. But when it comes to lip-reading as a means of teaching speech to deaf mutes the results are less satisfactory. The pupil can, by imitating the movements of the teacher's mouth, produce articulate sounds; but in very few cases does the effort not appear. The ordinary result is rather painful. The deaf mute speaks in a high key, gradually shrilling higher, the tone is harsh and monotonous; the speech is more like that of a well-trained parrot than that of a human being. The effect on the auditor is painful, and the speaker seems to fall back with relief upon the finger language. Nevertheless, when lip-reading is at its best it does much to relieve the depressing isolation in which the deaf, more even than the blind, seem to dwell.

How much is spent on the education of the defective classes in Britain we do not know, but the admirable Education Bureau of the United States gives full statistics of the subject. From these it appears that there are 48 public boarding institutions for the deaf in the United States, 13 public day schools, and two private schools. The pupils number 8,309, and the teachers 657. The total expenditure is over two million dollars, or about £400,000. The private schools are, of course, conducted on business principles, and show a profit; but in the public institutions dependent more or less on charity, the expenditure is a little greater than the receipts. In institutions for the blind, on the contrary, the receipts are larger by 100,000 dollars than the expenditure. It would be interesting to know if this result is due to the greater interest felt in the blind by the charitable public, or to the money earned by the inmates in the various trades—broom-making, mattress-making, &c.—which they are taught. The total number of institutions is 33, with 3,215 pupils, and 318 teachers, besides 120 instructors in music.

While these institutions are conducted on principles already almost decided, and methods proved by experience, homes and schools for the feeble-minded and the criminal are still largely tentative in nature. Hitherto with regard to the feeble-minded too much stress has been laid on those intellectual forms of education, which to those of weak brain are at once the most difficult, and the most useless. The art of reading is not knowledge; it is only the key to knowledge, and to those who will never use that key, who are fundamentally incapable of gathering truths from books, it is useless to present it. The kindergarten to begin with, leading on to manual training and simple industries, will make the feeble-minded happier as well as more useful to the community than any attempt to drill them in acquirements which to them have no meaning.

The education of criminal children is the most difficult of all. The mere question of gradation is almost insoluble. Age implies no equality. The boy, more thoughtless than wicked, who is sent to a reformatory for stealing apples, should hardly be made to associate for years with the apprentice of Fagan, old in crime if not expert in it. The more guilty will corrupt the comparatively innocent, who will leave the "reformatory" ten times worse than he entered it. The managers of the industrial and reformatory schools in the United States seem almost unanimous on two points, (1st) that grading should not be by age, but that some attempt should be made to classify the inmates by their merits. As in large institutions this is difficult, they recommend (2nd) that the cottage or family principle be adopted as far as possible; that is, that a few children only be together, so that a close personal supervision may be exercised. As, however, some talk of "families" of thirty children, one cannot but think the responsibilities of parenthood are too large. Still, even a brood of this size will not give full scope to the possibilities of mischief which sometimes develop large institutions. Another point on which all are agreed is that a definite sentence is a blunder. Children should be sent to the school for their entire minority, so that any good implanted may have time to take root, though it should be left to the discretion of the managers to free any who may seem to deserve it. Of course, in institutions of this nature industrial training must have a foremost place in order that pupils may go out to the world fit to earn their living as honest citizens. In some places contract-work of various kinds is done. This is remunerative, as it pays to some extent for the inmates' board, and therefore the officials are inclined to approve of it; but it is less desirable than technical training in various trades. And, in the end, to make a lad self-supporting for life is cheaper than merely to make him contribute to the cost of his own detention.

